# EHMTI-0147. Medication overuse headache and alexithymia: a controlled study

**DOI:** 10.1186/1129-2377-15-S1-D51

**Published:** 2014-09-18

**Authors:** G Sances, F Galli, M Caputi, E Guaschino, MG Cuzzoni, G Nappi, C Tassorelli

**Affiliations:** 1 Headache Science Center, C. Mondino National Neurological Institute, Pavia, Italy; 2 Psychology, Vita-Salute San Raffaele University, Milan, Italy

## Background

Alexithymia is a term used to describe a disorder where patients have difficulty in expressing their own feelings in words. The presence of Ax had been related to the occurrence of chronic pain, but poorly studied in headache. Noteworthy, the presence of Alexithymia has been linked to specific correlates at level of posterior cingulated cortex [1].

## Aim

The aim of this study is analyzing the construct of Alexithymia in MOH patients.

## Methods

A clinical sample of 105 MOH patients (27 M, 78 F, mean age 47.49+10.03) and 78 control subjects (28 M, 50 F, mean age 41.51+11.03) had been enrolled for the administration of the Toronto Alexithymia Scale.

## Results

Compared to controls, MOH showed significant values in the total score (t (181) = -4.706, p<001), in Factor 1 (Difficulties-in-identifying-feelings) (t(181)=-5.296,p<.001), Factor 2 (Difficulties-in-describing-feelings) of TAS-20 (t (181) = -1.999, p<.05) and a trend for Factor 3 (Outside-oriented-thought) (t(181)=-1.799, p=.099), without differences for gender. In MOH group, a pathological level of Ax is shown by 47% of patients (vs 15.4% of controls), a borderline level by 28.1% (vs 23.6%), and no-Ax by 24.9% (vs 61%).

## Conclusion

Alexithymia seems an important psychological factor involved in MOH, even if it is not clear which is the link with headache, but pathophysiological and therapeutic meanings should be considered in further studies.

No conflict of interest .
